# Sample-efficient identification of high-dimensional antibiotic synergy with a normalized diagonal sampling design

**DOI:** 10.1371/journal.pcbi.1010311

**Published:** 2022-07-18

**Authors:** Jennifer Brennan, Lalit Jain, Sofia Garman, Ann E. Donnelly, Erik Scott Wright, Kevin Jamieson

**Affiliations:** 1 Paul G. Allen School of Computer Science & Engineering, University of Washington, Seattle, Washington, United States of America; 2 Foster School of Business, University of Washington, Seattle, Washington, United States of America; 3 Department of Biomedical Informatics, University of Pittsburgh, Pittsburgh, Pennsylvania, United States of America; 4 Pittsburgh Center for Evolutionary Biology and Medicine, Pittsburgh, Pennsylvania, United States of America; University of Virginia, UNITED STATES

## Abstract

Antibiotic resistance is an important public health problem. One potential solution is the development of synergistic antibiotic combinations, in which the combination is more effective than the component drugs. However, experimental progress in this direction is severely limited by the number of samples required to exhaustively test for synergy, which grows exponentially with the number of drugs combined. We introduce a new metric for antibiotic synergy, motivated by the popular Fractional Inhibitory Concentration Index and the Highest Single Agent model. We also propose a new experimental design that samples along all appropriately normalized diagonals in concentration space, and prove that this design identifies all synergies among a set of drugs while only sampling a small fraction of the possible combinations. We applied our method to screen two- through eight-way combinations of eight antibiotics at 10 concentrations each, which requires sampling only 2,560 unique combinations of antibiotic concentrations.

## Author summary

Antibiotic resistance is a growing public health concern, and there is an increasing need for methods to combat it. One potential approach is the development of synergistic antibiotic combinations, in which a mixture of drugs is more effective than any individual component. Unfortunately, the search for clinically beneficial drug combinations is severely restricted by the pace at which drugs can be screened. To date, most studies of combination therapies have been limited to testing only pairs or triples of drugs. These studies have identified primarily antagonistic drug interactions, in which the combination is less effective than the individual components. There is an acute need for methodologies that enable screening of higher-order drug combinations, both to identify synergies among many drugs and to understand the behavior of higher-order combinations. In this work we introduce a new paradigm for combination testing, the normalized diagonal sampling design, that makes identifying interactions among eight or more drugs feasible for the first time. Screening *d* drugs at *m* different combinations requires *m* ⋅ 2^*d*^ samples under our design as opposed to *m*^*d*^ under exhaustive screening, while provably identifying all synergies under mild assumptions about antibiotic behavior. Scientists can use our design to quickly screen for antibiotic interactions, accelerating the pace of combination therapy development.

This is a *PLOS Computational Biology* Methods paper.

## 1 Introduction

Antibiotic resistance poses a clinical problem for which there are few available solutions. One promising strategy is the use of synergistic antibiotic pairings whose collective potency is greater than expected [1]. Commercially available examples include the antibiotics trimethoprim and sulfamethoxazole, which inhibit separate steps in the folate biosynthesis pathway [2], and quinupristin and dalfopristin, which both inhibit the ribosome [3]. Very few examples of synergistic combinations exceed two antibiotics [4], partly because the number of measurements required to detect multi-antibiotic synergy increases exponentially with the number of antibiotics tested. Exhaustively testing 10 concentrations of five antibiotics would require on the order of 10^5^ experiments, which limits the search space even when employing robotics to facilitate experimentation [5]. Scaling beyond five antibiotics is therefore impractical, and another approach is needed to explore the space of possible synergies.

Many methods have been developed to search this space. Much of the existing work focuses on identifying combinations with performance exceeding their “expected performance” under some *null model*, i.e., a prior belief about the effectiveness of antibiotics in combination. Measures of antibiotic performance include the absolute *amount* of cells (e.g., optical density after 18 hours) [6, 7], the *rate* at which cells grow [8], or the *energy released* by the cells [9]. The three most popular null models are Bliss [10], Highest Single Agent (HSA) [11], and Loewe [12]. Under the Bliss model, antibiotic effects combine probabilistically, so that combining antibiotic *A* at a concentration that yields a 50% reduction in growth with antibiotic *B* at a concentration that yields an 80% reduction in growth should result in a 90% reduction in growth (the probability of *A* *or* *B* independently killing the bacteria); if the combination has an efficacy greater than 90%, then it is called synergistic. In contrast, the HSA model posits that the effect of a drug combination is equivalent to the maximum of the effects of each drug used by itself. In the preceding example, *B* is the highest single agent, with an 80% individual reduction in growth, so any combined effect above 80% is considered synergy. Under the Loewe model, antibiotic *doses* combine linearly, so that combining antibiotic *A* at half of its minimum inhibitory concentration (MIC) and antibiotic *B* at half of its MIC should result in an inhibitory drug (this is consistent with the “sham combinations” principle, i.e., that half a unit of *A* plus half a unit of *A* makes a whole unit of *A*). If the combination is inhibitory at a lower concentration, it is considered synergistic under the Loewe model. We direct the reader to Foucquier and Guedj [13] for a comprehensive review of Bliss, Loewe, HSA, and other notions of synergy.

A complementary approach is to define an appropriate combination index (CI) on the space of antibiotic combinations at specific concentrations [11, 13, 14]. The *CI* of a dose combination measures a deviation from the null model, so that *CI* = 1 implies the combination follows the null model, and *CI* < 1 implies synergy. For *effect-based* null models, such as Bliss or HSA, the *CI* is naturally defined as the ratio of the prediction of the null model on a combination of drugs to the true effect of that combination. For *dose-effect models*, such as Loewe, the *CI* is defined relative to underlying isoboles of the same growth level. The Fractional Inhibitory Concentration Index (FICI) is the *CI* associated with Loewe synergy; an FICI of 1 means the drugs interact according to the Loewe Model [13]. For example, if a combination of drug *A* at $\frac{1}{4}$ of its MIC and drug *B* at $\frac{1}{2}$ of its MIC is inhibitory, then the FIC for this combination is $\frac{1}{4}+\frac{1}{2}=\frac{3}{4}$, and the FICI of *A* + *B* is therefore no greater than $\frac{3}{4}$. The FICI is interpreted against a standard scale, where values below 1 indicate synergy, while values between $\frac{1}{2}$ and 1 indicate “weak” synergy [15]. The FICI can also be viewed through a more clinically relevant lens as the minimum fractional concentration among all effective combinations. This perspective leads to a natural optimization problem: find the combination with the lowest normalized dose that is still effective.

Recent research has focused on identifying synergy among more than two drugs. When moving to higher order interactions, a distinction emerges between *total synergy*, which captures the combination’s performance relative to its individual components, and *emergent synergy*, which captures the incremental benefit over any subset of the combination; these notions have been defined relative to both the Bliss [5, 6, 16, 17] and Loewe [18] null models. However, a major challenge with identifying higher order interactions is the mounting evidence suggesting that the null models predicted by Loewe and Bliss poorly fit experimental data [7, 19]. In response, some authors have proposed *data-driven predictive models* trained only on pairs of drugs and then evaluated on three or more drugs, such as the dose model [7, 20], pairs model [8, 21], and the static λ score [18, 22, 23]. Given that they have no access to data from higher order combinations, predictive models effectively become null models for interactions between more than two drugs. This lets us characterize deviations from the predictive model as “emergent” antagonisms and synergies that arise from higher order effects not predictable by pairs.

Given a specific definition of synergy, a second major challenge is the high measurement burden of identifying synergistic combinations. For example, 10^8^ measurements are required to exhaustively test eight drugs at 10 concentrations each. Strategies to overcome exhaustive sampling fall into two categories: *parametric modeling* and *experimental design*. The former approach applies concepts from machine learning to build parametric models that can potentially predict all possible drug combinations accurately but can be learned using only a fixed number of parameters. Models of this form include the previously mentioned dose [20] and pairs [21] models, which explicitly assume no higher order interactions beyond pairs of drugs; mechanistic models, which use knowledge of the underlying drug targets [24] or gene expression data [25]; and the MAGENTA model [26], which leverages phenotypic information about the cell’s response to antibiotics.

The experimental design approach is a complementary strategy that reduces the amount of data to be collected, sometimes at the cost of data fidelity. For example, some studies severely restrict the number of doses per antibiotic combination [5] in order to exhaustively sample the space of possible combinations. Other studies employ *diagonal sampling*, where the relative proportions of the antibiotics remain fixed but the absolute quantities vary [7, 18, 27]. The diagonal sampling method has been proposed as a way to feasibly sample in higher dimensions and has been justified with the claim that the diagonal “provides the most information about the shape of the contour [phenotype isobole]” [18]. While the validity of diagonal sampling for Loewe synergy has received experimental support in some studies [22, 23], to date no work has rigorously justified its use or provided any guarantee about what kinds of synergies a diagonal design may or may not uncover. Absent such a rigorous justification, any study that fails to find synergy leaves open the possibility that synergy may still exist.

This work makes three major contributions: (1) we develop a novel CI, the Minimax Effective Concentration Index (MECI), which naturally extends the FICI and HSA models; (2) we present a theoretical framework for determining total and emergent synergies based on the MECI; and (3) we propose a new experimental design for provably identifying the MECI, called *normalized diagonal sampling* (NDS). The MECI has dual advantages over previously proposed metrics. It can be efficiently identified in high dimensions using the NDS design; under realistic assumptions about the behavior of antibiotics in combination, we prove that the NDS design finds the MECI with exponentially fewer samples than the full factorial experiment that samples all drugs at all combinations of concentrations. Finding the MECI of an 8-drug combination using 10 concentrations requires only 10 ⋅ 2^8^ ≈ 2500 samples, whereas finding the FICI in the same setting via the full factorial design would require an infeasible 10^8^ samples. The second advantage of the MECI is the flexibility it gives the experimentalist to capture clinically relevant drug combinations. The definition of the MECI includes a normalization factor for each drug, and the choice of normalization can be tailored to the goals of the synergy study at hand. We present two examples of the normalization factor in Section 2.3.

## 2 Methods

### 2.1 The Minimax Effective Concentration Index

In clinical practice the goal is to administer antibiotic combinations that are effective while avoiding high doses, which may cause adverse effects. Antibiotic doses are measured relative to various metrics, such as the strain-specific MIC or species-specific breakpoints, determined by bodies such as the European Committee on Antimicrobial Susceptibility Testing (EUCAST) [28]. In this section, we define the *MECI*, which captures the idea of avoiding high doses by minimizing the highest single antibiotic’s concentration (appropriately normalized) among antibiotic combinations that are “effective” at inhibiting growth. The precise definition of an “effective combination” is specified by the practitioner but could, for example, represent whether or not growth at a predefined time point remains below a pre-specified threshold.

Given a definition of an effective drug combination, we can now define the MECI. Let Ω = {1, 2, 3, …, *d*} index a set of *d* drugs, where drug *i* will be tested at *m* − 1 concentrations *χ*_*i*_ = {*x*_*i*,1_, *x*_*i*,2_, …, *x*_*i*,*m*−1_}. The concentrations of drug *i* are normalized to concentration *N*_*i*_ for analysis purposes so all drugs can be compared on a similar scale. In our experiments, we chose to test each drug at the same set of normalized ratios $\frac{x_{i,j}}{N_{i}}$.

Define $X\left(\Omega \right):=\prod_{i\in \Omega }\left(\chi_{i}\cup 0\right)$, the set of all possible combinations of all subsets of the *d* drugs in Ω at their *m* − 1 concentrations. A single x∈X(Ω) represents a single experimental condition, with *x*_*i*_ encoding the concentration of drug *i* (or *x*_*i*_ = 0 if drug *i* is absent). The set X(Ω) represents all *m*^*d*^ experimental conditions that would be tested in the full-factorial experimental design.

We define the **minimax concentration** of any x∈X(Ω) as the maximum normalized concentration among all drugs in the set Ω, i.e., ${\text{max}}_{i\in \Omega }\frac{x_{i}}{N_{i}}$. The **Minimax Effective Concentration Index (MECI)** is the smallest minimax concentration among all effective combinations x∈X(Ω). Specifically, the MECI of a set of drugs Ω can be expressed succinctly as the solution to an optimization problem:

$$\begin{array}{cc} MECI(\Omega ) & =\underset{x\in X(\Omega )}{\text{min}}\underset{i\in \Omega }{\text{max}}\frac{x_{i}}{N_{i}}\;\;\text{such}\;\text{that}\;\;x\;\text{is}\;\text{effective} \end{array}$$ (1)

The MECI({*i*}) of a single antibiotic *i* is the normalized concentration at which *i* is individually effective. For example, if *N*_*i*_ is the MIC of drug *i* and a drug is considered “effective” if it completely inhibits growth, then MECI({*i*}) = 1.

When screening for synergy, it is important to identify combinations where each antibiotic’s inclusion is justified by the effectiveness it brings to the combination. Indeed, synergies among lower order antibiotic combinations are likely to be detected in higher order combinations that include those same drugs; therefore, finding clinically useful combinations requires determining whether all antibiotics in a combination are necessary to achieve a synergistic effect. To this end, we develop two scores for interpreting whether a drug combination provides a meaningful improvement over its components: the Total Synergy Score (TSS) and the Emergent Synergy Score (ESS). The **TSS** measures the reduction in maximum concentration attainable by the combination as compared to each individual drug. For any combination of drugs Ω, it can be expressed mathematically as

$$\begin{array}{c} TSS(\Omega )=\frac{MECI(\Omega )}{{\text{min}}_{i\in \Omega }MECI\left(\left\{i\right\}\right)}. \end{array}$$ (2)

If the normalization *N*_*i*_ is the MIC of each drug, then the denominator min_*i* ∈ Ω_ *MECI*({*i*}) = 1, and the TSS is identical to the MECI. However, if the normalization can potentially be very far from the MIC, reporting the TSS ensures that a combination must improve upon its best single ingredient in order to be considered synergistic.

We further define the **ESS** to capture the improvement offered by the combination when compared to any of its subsets, following the intuition that the combination must significantly improve upon any subset of its ingredients to justify the use of additional drugs. The ESS is defined as

$$\begin{array}{c} ESS(\Omega )=\frac{MECI(\Omega )}{{\text{min}}_{\Omega^{\prime }\subset \Omega }MECI\left(\Omega^{\prime }\right)}. \end{array}$$ (3)

We observe that *TSS*(Ω) ≤ *ESS*(Ω) ≤ 1. An ESS score of 1 indicates that some proper subset of Ω has an MECI at least as small as Ω itself; this can occur in instances of indifference or antagonism. The TSS and ESS scores identify only synergy, not antagonism; for a discussion of antagonism, see Section 4.

**Fig 1:**
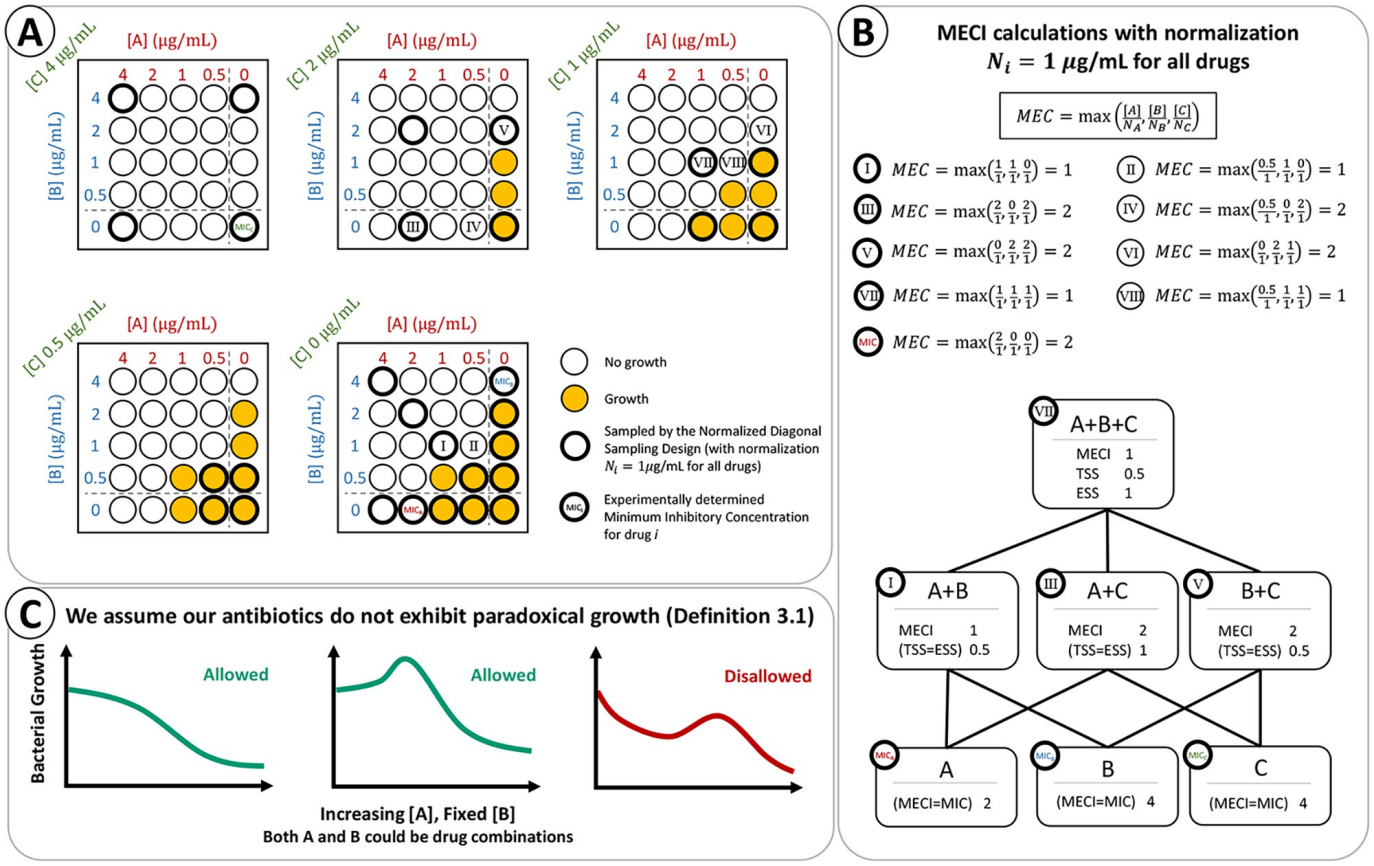
Calculation of the MECI, TSS, and ESS for a three-drug experiment. (A) Testing three drugs at four concentrations each could be performed exhaustively using a three-dimensional checkerboard assay, as depicted here. When the effectiveness measure is taken to be the absence of visible growth, the MEC can be calculated for each effective measurement in the checkerboard assay. (B) Computation of select MEC values from the checkerboard assay. The MECI of a drug combination is the minimum MEC among tested combinations. The MECI, TSS, and ESS of all subsets are computed according to their definitions, with normalization *N*_*i*_ = 1 *μ*g/mL for all drugs. The well in the upper-left corner of each combination’s calculated values witnesses the MECI of that combination; we see that the wells tested by the NDS design (shown with bold edges) are sufficient to identify the MECI. (C) If the behavior of the antibiotics satisfies Definition 3.1, we prove that the NDS design always identifies the MECI for every combination tested. We illustrate some examples of allowed and disallowed behavior of the measured response as a function of increasing some antibiotic (combination) *A* as the concentration of antibiotic (combination) *B* remains fixed.

While any ESS less than 1 indicates emergent synergy, we suggest as interpretive criteria that conclusions of **synergy** be restricted to combinations with an ESS of 0.25 or below, while combinations with an ESS between 0.25 and 1 should be interpreted as evidence of **weak synergy**. These interpretive criteria are consistent with the FICI interpretation of weak synergy when two drugs are tested according to the NDS design, since the cutoff FICI of 0.5 and the cutoff ESS of 0.25 both occur when two drugs are combined at $\frac{1}{4}$ of their MICs to yield an effective combination. Fig 1 illustrates the calculation of the MECI, TSS and ESS for a three-drug experiment.

### 2.2 Relationship to other metrics

For comparison to an established metric, we note that the FICI, like the MECI, can be written as the solution to an optimization problem. The FICI of combination Ω is given by

$$\begin{array}{cc} FICI(\Omega ) & =\underset{x\in X(\Omega )}{\text{min}}(\sum_{i\in \Omega }\frac{x_{i}}{MIC_{i}})\;\;\text{such}\;\text{that}\;\;x\;\text{is}\;\text{effective}. \end{array}$$ (4)

Note that the FICI differs from the MECI in two important ways. First, the FICI measures the amount of drug in each combination using its summed fractional concentration $\sum_{i\in \Omega }\frac{x_{i}}{MIC_{i}}$, whereas the MECI uses the maximum fractional concentration ${\text{max}}_{i\in \Omega }\frac{x_{i}}{N_{i}}$. Second, the FICI exclusively normalizes to the MIC of each drug, while the MECI allows flexibility in the choice of normalization.

We conclude by observing that the MECI corresponds to a dose-effect interpretation of the HSA model. Intuitively, the MECI minimizes the minimax concentration among effective combinations, where the minimax concentration is exactly the highest (normalized) concentration of a single agent. This connection is made precise in S2 Appendix.

### 2.3 Choice of normalization

The MECI provides flexibility in the choice of normalizing metric *N*_*i*_, which allows the experimentalist to tailor the choice of *N*_*i*_ to the goals of the investigation. In particular, different choices of *N*_*i*_ lead to different interpretations of synergy. For example, a natural choice of normalization *N*_*i*_ is the MIC for each drug *i*. With this choice, a low ESS or TSS indicates that the combination is effective at concentrations far below the individual drugs’ MICs, which aligns with the Loewe (and, to a lesser extent, Bliss) conceptions of synergy.

Another meaningful choice of *N*_*i*_ could be a strain-independent metric like the EUCAST breakpoint, which is the highest concentration at which the organism is considered sensitive to the antibiotic [28]. For example, when testing for synergy in a multi-drug-resistant strain, a clinically important goal could be to find a combination in which each drug is present below its breakpoint concentration, so that *MECI* ≤ 1.

We observe that it is possible for a drug combination to be synergistic with respect to one normalization *N*_*i*_ but not another, or for the strength of the interaction to depend upon the choice of normalization. As a result, it is important for the choice of *N*_*i*_ to reflect the scientific or clinical goal of the experiment and for this choice to be reported along with the synergy scores.

### 2.4 The normalized diagonal sampling design

We introduce the *NDS design*, an experimental design that samples all combinations of drugs in Ω at equal concentrations relative to the normalization *N*_*i*_. We begin with a set of ratios at which each drug will be tested, $\frac{x_{i}}{N_{i}}\in \left\{c_{1},c_{2},\ldots ,c_{m-1}\right\}$. For example, the ratios may be powers of two, *c*_*j*_ = 2^−*j*^, in which case the drugs are tested on a two-fold dilution gradient. The spacing and number of concentrations should be chosen to balance the resolution of the synergies detected with the number of experiments required, while testing a sufficient range of concentrations so that all drug combinations can be observed at both *effective* and *ineffective* concentrations. For all subsets Ω′ ⊆ Ω, the NDS design evaluates the combination Ω′ at all *m* − 1 normalized concentrations {*c*_1_ **1**, *c*_2_ **1**, …, *c*_*m*−1_ **1**}, where **1** represents a vector of all ones of size |Ω′|. In Section 3.1 we prove that, under mild assumptions about the behavior of antibiotic combinations, NDS provides strong theoretical guarantees on the detection of synergy. In particular, the design provably identifies the MECI from a collection Ω of *d* antibiotics without having to sample all $\left|X\left(\Omega \right)\right|=m^{d}$ possible combinations. Since the NDS design provably finds the MECI, high-dimensional antibiotic combination screens can be run with the confidence that if no synergies are identified, then none exist.

Exhaustive tests for synergy are conducted with checkerboard assays (see Fig 1) requiring *m*^*d*^ wells to screen *d* drugs at *m* concentrations each. The NDS design significantly reduces the required number of wells by testing along the “diagonal” − testing each combination with all drugs present at the highest concentration, then at the second-highest concentration, and so on. Under the NDS design, each of the 2^*d*^ drug combinations requires only *m* wells, for a total requirement of *m* ⋅ 2^*d*^ wells. For eight drugs and 10 concentrations per drug, this requires *m* ⋅ 2^*d*^ = 10 ⋅ 2^8^ = 2, 560 wells, or about twenty six 96-well plates. This is experimentally feasible, whereas *m*^*d*^ = 10^8^ wells (requiring approximately 10^6^ plates) is not.

### 2.5 Quantification of bacterial growth

Computing the MECI requires the analyst to specify an *effectiveness metric* that specifies whether the antibiotics tested were *effective* or *ineffective* at the given concentrations **x**. For our experiments, we considered an antibiotic combination *effective* in inhibiting bacterial growth if the area under the growth curve (AUGC) was less than a predefined threshold. The AUGC measures the area under the curve of optical density (at 600nm) over time after subtracting the background optical density reading. The area was approximated using a trapezoidal Riemann sum, taken in 15 minute increments over the 24 hours following inoculation. We also considered quantifying effectiveness using the maximum growth rate, computed as the maximum slope of a five-point moving average of the log optical density versus time curve; since this technique agreed with the AUGC, we did not include it in our results. Any measurement that captures the notion of antibiotic effectiveness could be used to compute the MECI.

### 2.6 Experimental conditions

Experiments were performed using the wild-type *E. coli* strain MG-1655 (NR-2653; BEI Resources, Manasses, VA, USA) in BBL Mueller Hinton II Broth (Cation-Adjusted) (BD Diagnostics, Spark, MD, USA). All experiments were performed in duplicate in 96 well plates and were fully randomized across well, plate and day of experimentation with the use of the OT-2 liquid handling robot (Opentrons, Brooklyn, NY, USA). In each well, antibiotics were diluted into 200 *μL* of media, then 50 *μL* of a 10^−4^ dilution of *E. coli* overnight culture, incubated at 37°C in BBL Mueller Hinton II Broth (Cation-Adjusted), was added. A fresh preparation of overnight culture was used for each day of experimentation. Plates were sealed with a gas-permeable sealing membrane (Breathe-Easy, Sigma-Aldrich, St. Louis, MO, USA) and incubated at 37°C for 24 hours, during which time optical density readings (600nm) were taken at 15 minute increments using a Biotek BioStack II coupled to a Biotek Epoch II Microplate Spectrophotometer. Plates were orbitally shaken for 15 seconds prior to each reading.

Eight antibiotics were chosen for their diversity of class and mechanism of action: ampicillin, aztreonam, ceftazidime, chloramphenicol, ciprofloxacin, gentamicin, trimethoprim, and tobramycin. Antibiotics were dissolved in water with the exception of aztreonam, ceftazidime and trimethoprim, which were dissolved in DMSO, and chloramphenicol, which was dissolved in ethanol. Table 1 identifies the classes of each antibiotic used, EUCAST susceptible breakpoints [28], American Society for Microbiology Abbreviation (ASM code; https://journals.asm.org/journal/aac/abbreviations), and MICs determined experimentally.

**Table 1:** The eight antibiotics chosen for our studies, including EUCAST susceptible breakpoints [28] and experimentally determined MICs for *E. coli* strain MG-1655.

Antibiotic	ASM Code	Product Number	Antibiotic Class	EUCAST Susceptible Breakpoint (*μ*g/mL)	MIC (*μ*g/mL)
Ampicillin	AMP	BP1760 (Fisher Scientific)	*β*-lactam (penicillin)	8	16
Aztreonam	ATM	15151 (Chem-Impex)	*β*-lactam (monobactam)	1	0.25
Ceftazidime	CAZ	AC461730050 (Acros Organics)	*β*-lactam (cephalosporin)	1	0.25
Chloramphenicol	CHL	C0378 (Sigma-Aldrich)	Amphenicol	8	8
Ciprofloxacin	CIP	199020 (MP Biomedicals)	Quinolone	0.25	0.015625
Gentamicin	GEN	00149 (Chem-Impex)	Aminoglycoside	2	0.5
Trimethoprim	TMP	92131 (Sigma-Aldrich)	Antifolate	4	0.25
Tobramycin	TOB	455430010 (Acros Organics)	Aminoglycoside	2	0.5

## 3 Results

The full-factorial sampling design is intractable beyond a small number of antibiotics, which motivates the need for a more efficient design. We begin with a proof of correctness for the NDS design, showing that it identifies the MECI using significantly fewer measurements than the full factorial design. The NDS design samples along the diagonal in multi-antibiotic concentration space, letting us find all synergies among combinations of up to eight antibiotics at 10 concentrations with only 2,560 samples. This scale of experiment can be effectively pipetted by hand or using robotics, as we did here. Using robotics enabled us to perform complete pipetting randomization, minimizing plate position effects. In combination, the NDS design and robotics let us confidently identify synergies while mitigating the influence of experimental artifacts that complicate large-scale experiments.

### 3.1 The NDS design provably finds the MECI

We now show that the NDS design provably identifies the MECI of a set of drugs using significantly fewer samples than the full factorial design. Since the NDS design for a set of drugs Ω also involves performing the NDS design for all sets Ω′ ⊂ Ω, it correctly identifies the MECI, TSS, and ESS for each Ω′ ⊆ Ω.

The proof begins with an assumption that adding an antibiotic *A* to a fixed concentration of antibiotic *B* does not exhibit *paradoxical growth*, that is, once increasing the amount of *A* reduces the level of the measured response (e.g., growth), further increasing *A* cannot increase the response. Next, we observe that a fixed-ratio combination of drugs itself behaves like an antibiotic, with its own dose response curve and its own interactions with other (combination) antibiotics. This perspective lets us extend the assumption to the general case where *A* and *B* are themselves antibiotic combinations, stated formally in Definition 3.1. When the effectiveness measure in the definition of MECI behaves according to Definition 3.1, we can declare many combinations ineffective without ever measuring them because they lie between points already measured to be ineffective. Without such an assumption, we would have no way to know whether a point *x* is effective without testing it. The formal theorem statement is provided in Theorem 3.2; the proof is available in S1 Appendix.

**Definition 3.1** (Absence of paradoxical growth). *Let* Ω *be a set of antibiotics. Let the vector* $x_{0}\in R_{\geq 0}^{\left|\Omega \right|}$ *represent a fixed background concentration of antibiotics to which we add increasing amounts of another antibiotic combination* $x\in R_{\geq 0}^{\left|\Omega \right|}$. *We say the set of drugs* Ω ***does not exhibit paradoxical growth** if, for all* *c*_3_ > *c*_2_ > *c*_1_ ≥ 0, *the response* $r:R_{\geq 0}^{\left|\Omega \right|}\to R$ *satisfies*

$$\begin{array}{c} r\left(x_{0}+c_{2}x\right)<r\left(x_{0}+c_{1}x\right)\Rightarrow r\left(x_{0}+c_{3}x\right)\leq r\left(x_{0}+c_{2}x\right). \end{array}$$ (5)

Fig 1C illustrates our assumption by showing several allowed and disallowed shapes of the dose-response curve as some combination *A* is added to the base combination *B*. We specifically note that this assumption does not preclude so-called “hyper-antagonism,” where the addition of *A* to *B* yields a less effective response than *B* alone. Such behavior is allowed under our assumption as long as the following condition holds: once increasing concentration of *A* starts reducing the response *r*, further increasing the concentration continues to reduce the response.

How plausible is the assumption of non-paradoxical growth? When antibiotic effectiveness is measured in a broth dilution assay, as in our experiments, we are aware of no published evidence of paradoxical growth. When effectiveness is measured using a survival assay, in which bacteria are first treated with antibiotics for a specified time and then the culture is grown in the absence of antibiotics, paradoxical growth is known as the *Eagle effect*, first observed by Eagle and Musselman [29] and reviewed in [30]. If any antibiotic or combination in an experiment displayed the Eagle effect, then the NDS design could fail to identify synergies if drug effectiveness were quantified using a survival assay. To further support our assumption of non-paradoxical growth, we tested 100 randomly chosen ratios of antibiotics against fixed background combinations of antibiotics, the precise setting in Definition 3.1. S3 Appendix shows the results of these experiments, in which no paradoxical growth was observed.

Suppose we define a combination of drugs at a given concentration **x** as *effective* whenever the response falls below some threshold (*r*(**x**) ≤ *t*). Then, as long as the response behaves according to Definition 3.1, we can identify entire regions of the antibiotic combination space as *ineffective* using only measurements on the boundary of the space. This leads to our main result: the correctness of the NDS design in the absence of paradoxical growth.

**Theorem 3.2** *Assume the set of drugs* Ω *does not exhibit paradoxical growth (Definition 3.1). Then, the NDS design applied to* Ω *identifies MECI*(Ω′) *for all* Ω′ ⊆ Ω.

The proof of Theorem 3.2 is deferred to S1 Appendix. We observe that the NDS design provably identifies *MECI*(Ω) with significantly fewer samples than the naive full factorial design: the full factorial design requires *m*^*d*^ samples, while the NDS design requires only *m* ⋅ 2^*d*^.

### 3.2 Experimental results

#### 3.2.1 Identification of emergent effects relative to breakpoint

Using the NDS design, we performed experiments to screen all 2^8^ combinations of the 8 drugs shown in Table 1. The set of drugs was chosen to cover a wide range of mechanisms among drugs with defined EUCAST breakpoints for *Enterobacterales*.

Effectiveness was specified as complete inhibition of growth, measured by AUGC (as described in Section 2.5). The normalization constant *N*_*i*_ for the first experiment, used for both the NDS design and for calculating the TSS and ESS, was the EUCAST susceptible breakpoint [28] for *Enterobacterales*. As motivated in Section 2.3, normalization relative to the breakpoint provides a strain-independent measure of antibiotic interactions, which may be more relevant to how drugs are prescribed in combination clinically. We tested concentrations across 10 steps of a two-fold dilution gradient so that any interactions could be identified within a power of two.

**Fig 2:**
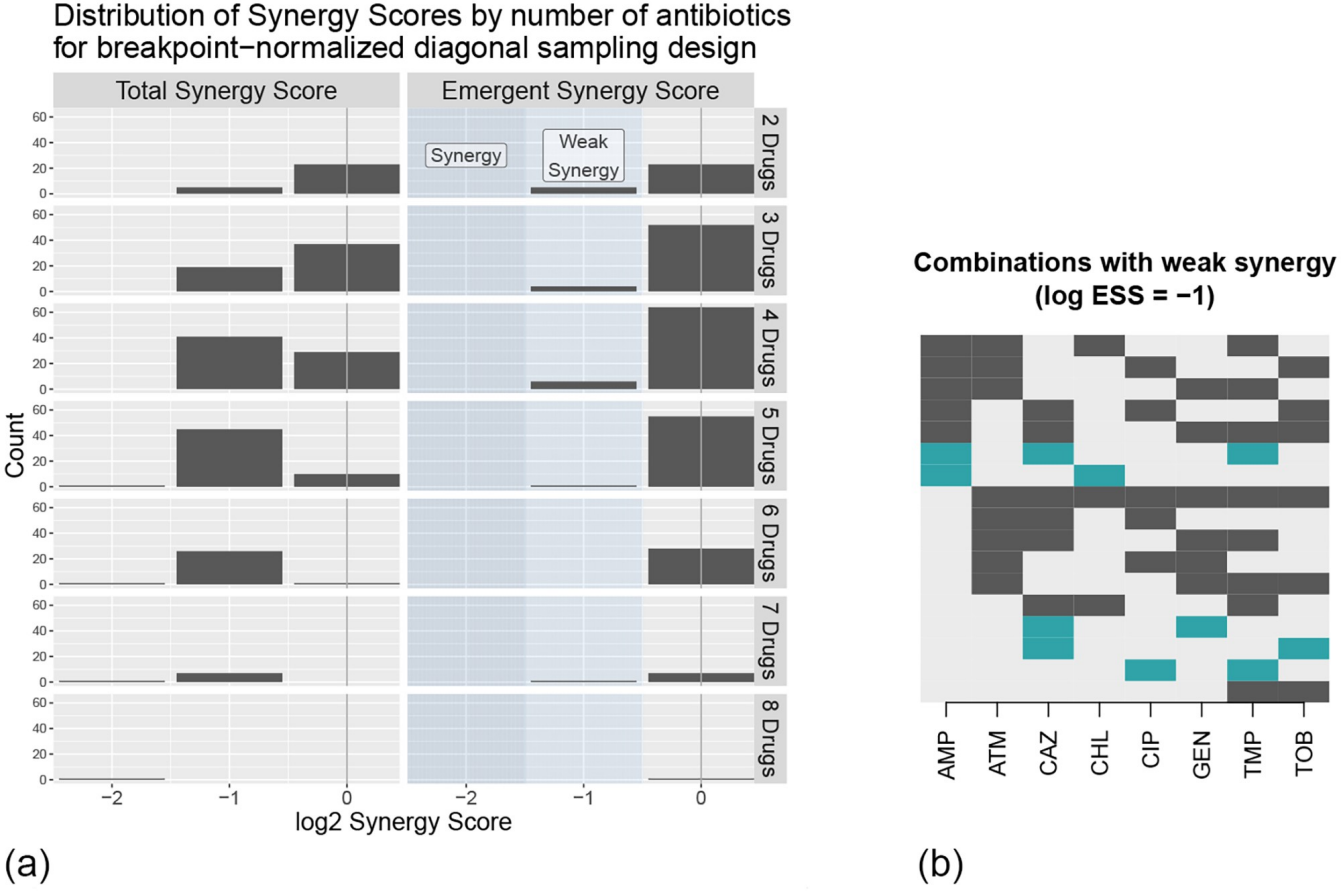
Results from the breakpoint-normalized experiment. (a) Distribution of TSS and ESS scores across all 2^8^ different breakpoint-normalized combinations of the eight antibiotics in Table 1, separated by the number of drugs in the combination. (b) Representations of the 17 drug combinations exhibiting weak synergy. Each row represents one combination; dark shades (black and blue) indicate presence of the drug, while light gray indicates absence. Black represents combinations that are weakly synergistic according to the breakpoint normalization but not the MIC normalization (next section), while blue shows the five combinations that exhibited weak synergy according to both normalizations.

We computed MECI, ESS and TSS for each combination of two through eight antibiotics. Histograms of the ESS and TSS scores across all combinations, plotted by the number of drugs combined, are shown in Fig 2A (recall that a lower ESS score indicates greater synergy, and a log_2_ ESS score of 0 indicates the absence of synergy). Among all 2^8^ combinations tested, no combination met our synergy threshold of a log_2_ ESS of −2 or lower (4-fold synergies), although several combinations exhibited weak synergy with a log_2_ ESS of −1. This indicates only weak synergy among all combinations of these eight antibiotics, consistent with previous studies that found synergy to be rare [5, 22, 23, 31]. As anticipated, the most commonly reported log_2_ emergent effect was zero. Fig 2B shows the makeup of the combinations exhibiting weak synergy. Full data, including the ESS and TSS of each of the 2^8^ combinations and a Loewe analysis showing the absence of strong Loewe synergy along the diagonals tested, is available in S4 Appendix.

We emphasize that our results come with strong guarantees under the assumption that the drugs do not exhibit paradoxical growth. Since our experiments were conducted according to the NDS design, Theorem 3.2 *guarantees* that, among these eight drugs, no combination has a log_2_ ESS score of −2 or less when ESS measures synergy relative to the EUCAST breakpoint. In particular, the result guarantees that performing the full factorial experiment would result in the same ESS and TSS scores that we found with the NDS design. If the goal is to minimize the maximum amount of drug applied relative to the EUCAST breakpoint, we therefore conclude that no significant gains are possible by combining this set of drugs against the strain we tested and under our experimental conditions.

#### 3.2.2 Identification of emergent effects relative to MIC

When compared to earlier methods such as the FICI, an important advantage of our synergy screening method is the flexibility provided by the choice of the normalizing constant *N*_*i*_. To demonstrate the utility of normalization, we repeated the preceding experiment but with all concentrations normalized to the MIC of the individual drugs (listed in Table 1). Normalizing to the MIC means that all drugs are combined at similar points along their dose-response curves, which we might expect to result in larger interactions. It also has the benefit of mirroring the FICI synergy screen, where the “fractional concentration” of a combination is always measured relative to the MICs of the individual antibiotics. For this reason, the MIC-normalized experiment is more directly comparable to previous synergy screening techniques.

**Fig 3:**
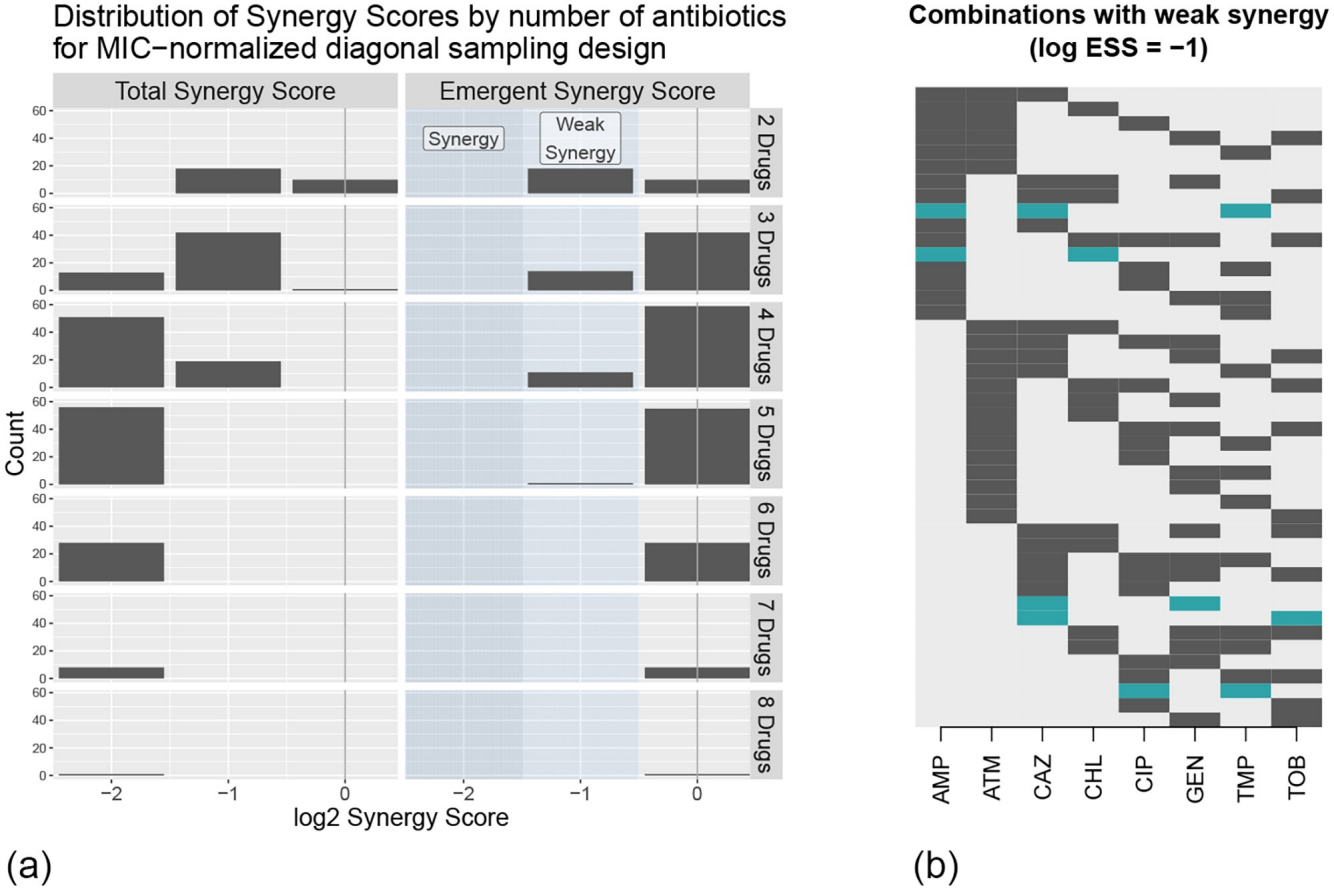
Results of the MIC-normalized experiment. (a) Distribution of TSS and ESS scores across all 2^8^ different MIC-normalized combinations of the eight antibiotics in Table 1, separated by the number of drugs in the combination. (b) Representations of the 44 drug combinations exhibiting weak synergy; combinations that were also weakly synergistic under the breakpoint normalization are shown in blue. Each row represents one combination; black/blue indicates presence of the drug, while gray indicates absence.

We computed the MECI, TSS and ESS for each combination of antibiotics and show the results in Fig 3. We see that the most common log_2_ ESS score is again 0, and that the lowest observed log_2_ ESS score is −1. The lack of strong synergy remains consistent with previous literature showing that synergy is rare [5, 22, 23, 31]. Again, we emphasize that the NDS design provides a strong guarantee in the absence of paradoxical growth: not only did we not find any combinations with log_2_ ESS of −2 or lower, we also know that there is no ratio at which the drugs can be combined that will exhibit an ESS of this magnitude. Fig 3B shows the combinations exhibiting weak synergy.

Compared to the breakpoint-normalized experiment, we observe that the results of the MIC-normalized experiment show more weak synergies and more negative log_2_ TSS scores. One possible explanation is that the breakpoint-normalized experiment combined drugs at concentrations with very different individual effectiveness. For example, ciprofloxacin has a breakpoint that is four steps above its MIC for this strain. As a result, combinations of ciprofloxacin and any other drug at concentrations several steps below the breakpoint may behave like ciprofloxacin alone, resulting in a log_2_ TSS of zero.

## 4 Discussion

We developed a novel scoring function to identify antibiotic interactions, the MECI, which applies the principles of the HSA model to determine whether an antibiotic combination is synergistic. We extend the HSA model into higher dimensions from both a theoretical perspective, developing a framework for identifying total and emergent synergies, and from a practical perspective, introducing a novel experimental design that provably identifies all total and emergent synergies. Applying our methodology to study combinations of eight antibiotics yielded no clinically relevant synergies, which we define as emergent synergies with a four-fold decrease or more in concentration when compared to their best subset. The strong theoretical guarantees of our sampling scheme allow us to conclude that there is no clinically relevant synergy among these eight drugs under the experimental conditions and normalization methods we considered, even though we sampled only a small fraction of the possible space of concentrations.

The MECI also has clinical relevance aside from the classification of synergistic combinations. When drug combinations are prescribed in clinical practice, each individual drug is typically administered at its standard dose. If the MECI is normalized relative to that standard dose, then an MECI <1 indicates that the drugs can each be administered at doses less than their standard dose while still remaining effective. In addition, if NDS is used to identify the MECI and paradoxical growth is absent, then there is no effective combination at doses less than the experimentally determined MECI.

We emphasize that the theoretical guarantees of our method apply only when the set of antibiotics exhibits non-paradoxical growth, which we define as the display of unimodal dose-response curves along any constant-ratio drug combination in the presence of any fixed background drug combination. Our assumption of non-paradoxical growth may be violated when the antibiotics exhibit the Eagle effect and the response is measured using a survival assay, or if higher order combinations of drugs behave very differently from our intuition based on the one- and two-drug settings. We further emphasize that these guarantees only apply to synergy as determined by the MECI, and that our design is not guaranteed to identify Loewe or Bliss synergies.

While the NDS design provably identifies all synergies relative to a given normalization, it is limited in that it cannot provably identify all antagonisms. Recall that the MECI quantifies the smallest minimax concentration among drug combinations measured to be effective. Another perspective on this optimization problem is to consider multiple rays representing different concentration ratios (see Fig 4A of [13]), which could be considered different “diagonals,” in the language of diagonal sampling. The MECI minimizes the minimax effective concentration along each of those rays. An analogous definition of *antagonism* from this perspective would be a new CI that *maximizes* the minimax concentration of *ineffective* combinations among these rays. Total and emergent antagonism could then be defined analogously to their definitions for synergy. Unfortunately, the NDS design’s theoretical guarantees extend only to minimizing the minimax effective concentration (identifying the greatest synergy), not to maximizing the minimax ineffective concentration (identifying the greatest antagonism). The difference is due to an asymmetry in Definition 3.1; it is possible for the response to increase and then decrease as antibiotic *B* is added to a fixed concentration of *A* (so-called *hyperantagonism*), but it is not possible for the response to decrease and then increase (paradoxical growth). Consequently, while an ESS of 1 indicates a lack of synergy, an ESS of less than 1 does not rule out the existence of antagonism in some part of the antibiotic combination space. Notwithstanding this limitation, we believe synergies are typically the most clinically relevant, so the inability to find antagonisms is therefore not too limiting in practice.

The NDS design makes provably identifying high-order interactions experimentally feasible for the first time. With this new method, it is now possible to screen for interactions among more antibiotics, across additional strains, and on multiple media, providing information on whether synergies are conserved across these variables. The NDS design is simple to implement: given a definition of antibiotic normalization (e.g., MIC), it samples along the diagonals in multi-antibiotic space. We conclude by noting that the interaction among biologically active agents is not only of interest for antibiotics, but also for many other biologically active agents, such as immunosuppressants [27], environmental toxins [32], anesthetics [33], and anticancer drugs [20, 34–36]. Whenever the absence of paradoxical growth can be assumed, the NDS design can be applied to identify synergy in high dimensions.

## Supporting information

S1 Appendix Proof of Theorem 3.2. (PDF)

S2 Appendix Connection between the Minimax Effective Concentration Index and the Highest Single Agent model. (PDF)

S3 Appendix Evidence of Non-Paradoxical Growth. (PDF)

S4 Appendix Loewe analysis of breakpoint- and MIC-normalized experiments. (PDF)

S1 Data and Code Dataset for the breakpoint- and MIC-normalized experiments, as well as code to reproduce the anlayses in this paper. (ZIP)

S1 Text List of contents of S1 Data and Code. (PDF)
