# Supplementary material for: Sample-efficient identification of high-dimensional antibiotic synergy with a normalized diagonal sampling design
Source: PLoS Comput Biol. 2022 Jul 18;18(7):e1010311. doi: 10.1371/journal.pcbi.1010311 (PMC9333450; doi:10.1371/journal.pcbi.1010311)
Supplement: S1 Appendix — (PDF) [file pcbi.1010311.s001.pdf]

Proof of Theorem 3.2

We begin by rewriting Definition 3.1 in an easier to manipulate form.

Proposition 1. *Suppose the set of drugs Ω does not display paradoxical growth, as defined in Definition 3.1, and let $\mathbf{x_0}, \mathbf{x_1} \in \mathcal{X}(\Omega)$ be two antibiotic combinations of drugs in Ω at specified concentrations. If $\mathbf{x_1} \geq \mathbf{x_0}$ coordinatewise, and both $\mathbf{x_0}$ and $\mathbf{x_1}$ display a response above some threshold t (so that $r(\mathbf{x_1}) \geq t$ and $r(\mathbf{x_0}) \geq t$), then any convex combination of the two points also has response above the threshold:*

$$r(\lambda \mathbf{x_0} + (1-\lambda)\mathbf{x_1}) \geq t \ \forall \lambda \in (0,1). \tag{6}$$

Proof. We begin by defining $\mathbf{x_\Delta} =: \mathbf{x_1} - \mathbf{x_0} \in \mathbb{R}_{\geq 0}^{|\Omega|}$, which itself can be viewed as its own antibiotic combination. Then, we have

$$\begin{aligned} r(\lambda \mathbf{x_0} + (1-\lambda)\mathbf{x_1}) &= r(\lambda \mathbf{x_0} + (1-\lambda)(\mathbf{x_\Delta} + \mathbf{x_0})) & (7)\\ &= r(\mathbf{x_0} + (1-\lambda)\mathbf{x_\Delta}) & (8)\\ &= r(\mathbf{x_0} + c\mathbf{x_\Delta}) & (9) \end{aligned}$$

for $c \in (0,1)$. Next, we have a case analysis on the relationship between $r(\mathbf{x_0} + c\mathbf{x_\Delta})$ and $r(\mathbf{x_1})$.

Case 1: $r(\mathbf{x_0} + c\mathbf{x_\Delta}) \geq r(\mathbf{x_1})$. In this case, we apply $r(\mathbf{x_1}) \geq t$ to conclude that $r(\lambda \mathbf{x_0} + (1-\lambda)\mathbf{x_1}) = r(\mathbf{x_0} + c\mathbf{x_\Delta}) \geq t$, as desired.

Case 2: $r(\mathbf{x_0} + c\mathbf{x_\Delta}) < r(\mathbf{x_1})$. In this case, we begin with the contrapositive of Assumption 3.1 with $\mathbf{x} = \mathbf{x_\Delta}$, $c_2 = c \in (0,1)$, $c_3 = 1$ and $c_1 = 0$. With these assignments, the contrapositive is

$$r(\mathbf{x_1}) > r(\mathbf{x_0} + c\mathbf{x_\Delta}) \implies r(\mathbf{x_0} + c\mathbf{x_\Delta}) \geq r(\mathbf{x_0}). \tag{10}$$

The hypothesis of the implication is satisfied, so we conclude that

$$r(\mathbf{x_0} + c\mathbf{x_\Delta}) \geq r(\mathbf{x_0}) \geq t \tag{11}$$

as desired. □

With this proposition in hand, we are now ready to prove our main theorem:

Proof of Theorem 3.2. Let $\{c_j\}_{j=1}^m$ denote the N_i-normalized concentrations tested under the NDS design. Let c_{j^*} denote the MECI identified by the design, with $j^* \in \{1, \ldots, m\}$.

To prove the theorem, we will leverage Proposition 1 to show that Assumption 3.1 implies that all combinations of the $d = |\Omega|$ drugs at concentrations with MEC less than c_{j^*} are ineffective, and therefore c_{j^*} is also the MECI among all m^d combinations. Note that, if the MECI corresponds to the smallest sampled concentration $j^* = 1$, we are done, because there is only one combination with MEC c_1, and the NDS design samples it. Therefore, in what follows, we will assume $j^* > 1$.

We will now show that the hypercube of side length c_{j^*-1}, extending from the origin to $c_{j^*-1}\mathbf{1}$, contains only ineffective points. To see this, first note that, under normalized diagonal sampling, all the vertices of this hypercube have been sampled. Next, observe that the vertices must all have come up "ineffective," or else c_{j^*} would not be the minimum-norm effective point. It remains to show that our assumption implies that all other points in the hypercube are ineffective.

By Assumption 3.1 and Proposition 1, we know that if $\mathbf{x_1} \geq \mathbf{x_2}$ componentwise and both are ineffective, then $\lambda\mathbf{x_1} + (1-\lambda)\mathbf{x_2}$ is also ineffective for all $\lambda \in [0,1]$. We will apply this rule recursively d times, to show that the entire hypercube is ineffective.

For the first step, we show that the one-dimensional edges of the hypercube are ineffective, by noting that every point on an edge is a convex combination of two points on the vertices of the cube, and therefore is ineffective. Next, we take the convex combination of all vertices and edges, which shows that the two-dimensional faces are all ineffective. We continue with this argument d times, concluding that all points with MEC less than c_{j^*} must be ineffective. This confirms that the MECI among all m^d possible combinations is the same as the MECI found with the diagonal sampling scheme. □
