# Supplementary material for: Sample-efficient identification of high-dimensional antibiotic synergy with a normalized diagonal sampling design
Source: PLoS Comput Biol. 2022 Jul 18;18(7):e1010311. doi: 10.1371/journal.pcbi.1010311 (PMC9333450; doi:10.1371/journal.pcbi.1010311)
Supplement: S2 Appendix — (PDF) [file pcbi.1010311.s002.pdf]

Connection between the Minimax Effective Concentration Index and the Highest Single Agent model

In this section, we justify our claim that our MECI gives a dose-effect version of the Highest Single Agent model. To see this it is worth taking an isobologram perspective [2, 1]. For concreteness focus on the case of two drugs A, B, and a fixed effect of interest, for example the OD10h. Assume we are given a specific value of the response E, let $x_A^*(E), x_B^*(E)$ be the minimal doses which achieve this value for each drug individually. Dose-effect models describe the set of points in dose space which should also have an effect E. The MECI naturally gives rise to a null model that posits that the corresponding isobole for a combination of drugs with effect E is the set $\{(x_A, x_B) | \max\{\frac{x_A}{x_A^*(E)}, \frac{x_B}{x_B^*(E)}\} = 1\}$. This is in contrast to the Loewe model considers the isobole $\frac{x_A}{x_A^*} + \frac{x_B}{x_B^*} = 1$.

Though dose models do not directly specify the effect of a combination of drugs directly, the effect can be imputed from knowledge of all possible isoboles. More precisely, given the set of isoboles corresponding to the entire range of possible effects E, we can determine for any given concentration (x_A, x_B) what the effect should be - namely the corresponding of the isobole passing through that point. Concretely for the Loewe model to find the expected effect at (x_A, x_B) we would need to find x'_A and x'_B both with the same effect so that the line $x_A/x'_A + x_B/x'_B = 1$ passes through x_A, x_B. In absence of dose responses for every possible concentration of A, B it is unclear how to find this quantity. In the case of the MECI, it is sufficient to know the effect of x_A and x_B individually to determine the isobole. Namely the effect at any point is just $\max\{r(x_A), r(x_B)\}$. This is effectively the HSA model on effects[2]. We note however that the MECI is different from the CI you would get by using the HSA effect model[2] since it is defined on concentrations relative to x_A^*, x_B^* not on the effect directly.

References

[1] Morris C Berenbaum. “Synergy, additivism and antagonism in immunosuppression. A critical review.” In: *Clinical and experimental immunology* 28.1 (1977), p. 1.

[2] Julie Foucquier and Mickael Guedj. “Analysis of drug combinations: current methodological landscape”. In: *Pharmacology research & perspectives* 3.3 (2015), e00149.
