# Supplementary material for: Sample-efficient identification of high-dimensional antibiotic synergy with a normalized diagonal sampling design
Source: PLoS Comput Biol. 2022 Jul 18;18(7):e1010311. doi: 10.1371/journal.pcbi.1010311 (PMC9333450; doi:10.1371/journal.pcbi.1010311)
Supplement: S3 Appendix — (PDF) [file pcbi.1010311.s003.pdf]

Evidence of Non-Paradoxical Growth

Central to our results is the absence of paradoxical growth, as given in Definition 3.1. Exhaustively verifying this assumption for all background combinations $\mathbf{x}_0$ and additive combination $\mathbf{x}$ would be as difficult as exhaustively searching the entire d-dimensional drug space, which is experimentally intractable. However, we can spot-check this assumption by measuring dose-response curves for various pairs of $(\mathbf{x}_0, \mathbf{x})$ and verifying that we observe no paradoxical growth.

We tested 100 randomly chosen pairs of $(\mathbf{x}_0, \mathbf{x})$, each representing a different (off-diagonal) ray in high-dimensional antibiotic space. The 100 pairs we tested represent less than 0.1% of the possible $(\mathbf{x}_0, \mathbf{x})$ combinations; testing more pairs would add confidence to our belief in non-paradoxical growth, but at the expense of additional experimental effort and resources. To select the background concentration vector $\mathbf{x}_0$, we first chose to include or exclude each of the 8 drugs with probability $\frac{1}{2}$. The concentration of included antibiotics was then selected as $\frac{1}{4}$ the MIC of those drugs in combination, as measured during our MIC-normalized experiment. This ensured that the background concentration was itself "ineffective," so that the dose-response curve of $\mathbf{x}_0 + c\mathbf{x}$ gave a nontrivial dose-response curve. Next, we randomly selected a concentration vector $\mathbf{x}$. Included antibiotics were then randomly assigned to be included at either their MIC, $\frac{1}{2}$MIC, or $\frac{1}{4}$MIC. This ensured that we were measuring "off-diagonal" rays in high-dimensional space, which is where we might be concerned about finding paradoxical growth. Note that a drug could be included in both the background set $\mathbf{x}_0$ and the additive set $\mathbf{x}$.

For each choice $(\mathbf{x}_0, \mathbf{x})$, we measured eight concentrations, from six 2-fold steps below the initially chosen concentration of $\mathbf{x}$ to two 2-fold steps above. Measurements were taken in three replicates.

Figure A shows the 100 dose-response curves generated from these experiments. Curves were generated by taking the average of three replicates at each concentration, then smoothed using a Gaussian kernel (bandwidth $\sigma = 1$) to reduce noise (this smoothing operator is less aggressive than the typical 3-point moving average used in, e.g., Katzir et al. [1]). We observe that, among the $(\mathbf{x}_0, \mathbf{x})$ tested, none display paradoxical growth (where paradoxical growth would be evidenced by a curve with AUGC that decreases then increases as the concentration increases, as in panel C of Figure 1). This evidence supports the assumption of non-paradoxical growth for our set of antibiotics.

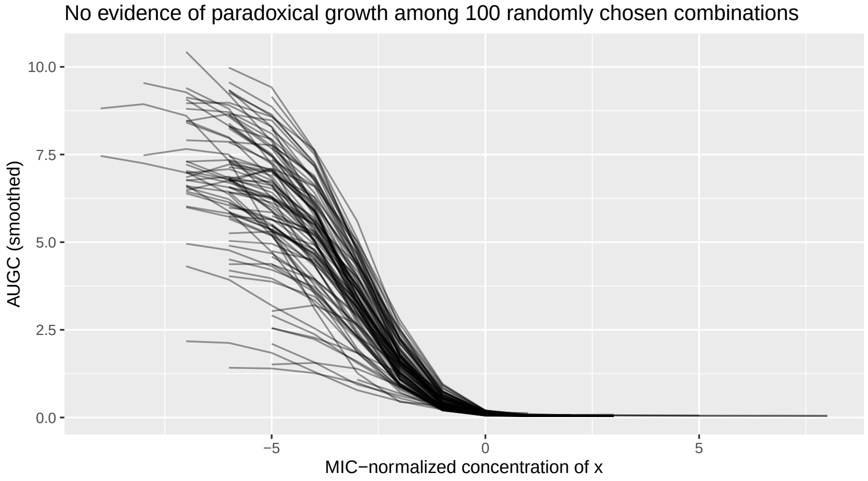

Figure A: Dose response curves for 100 randomly chosen baseline/additive pairs $(\mathbf{x}_0, \mathbf{x})$. Curves are translated to reach their MIC at the same point on the graph, to aid legibility. We observe no paradoxical growth; in particular, no curve shows a pattern of decreasing then increasing AUGC as the concentration increases.

References

[1] Itay Katzir et al. "Prediction of ultra-high-order antibiotic combinations based on pairwise interactions". In: *PLoS computational biology* 15.1 (2019), e1006774.
