# Supplementary material for: Sample-efficient identification of high-dimensional antibiotic synergy with a normalized diagonal sampling design
Source: PLoS Comput Biol. 2022 Jul 18;18(7):e1010311. doi: 10.1371/journal.pcbi.1010311 (PMC9333450; doi:10.1371/journal.pcbi.1010311)
Supplement: S4 Appendix — (PDF) [file pcbi.1010311.s004.pdf]

Loewe Analysis

In this section we provide a Loewe analysis of our experimental data, for both the breakpoint-normalized and MIC-normalized diagonal designs. To compute the Loewe score for an N drug combination at concentrations $x_1, \ldots, x_N$, we compute the sum of the individual MIC-normalized concentrations:

$$\sum_{i=1}^{N} \frac{x_i}{\mathrm{MIC}_i}$$

where MIC_i is the MIC of drug i alone. Figure A shows the distribution of Loewe scores for the breakpoint- and MIC-normalized experiments. Interpretation of synergy and antagonism follow the guidelines found in Table 4 of Chou [1]. Note that antagonism is common while synergy is rare, and no combination attained "strong" or "very strong" synergy.

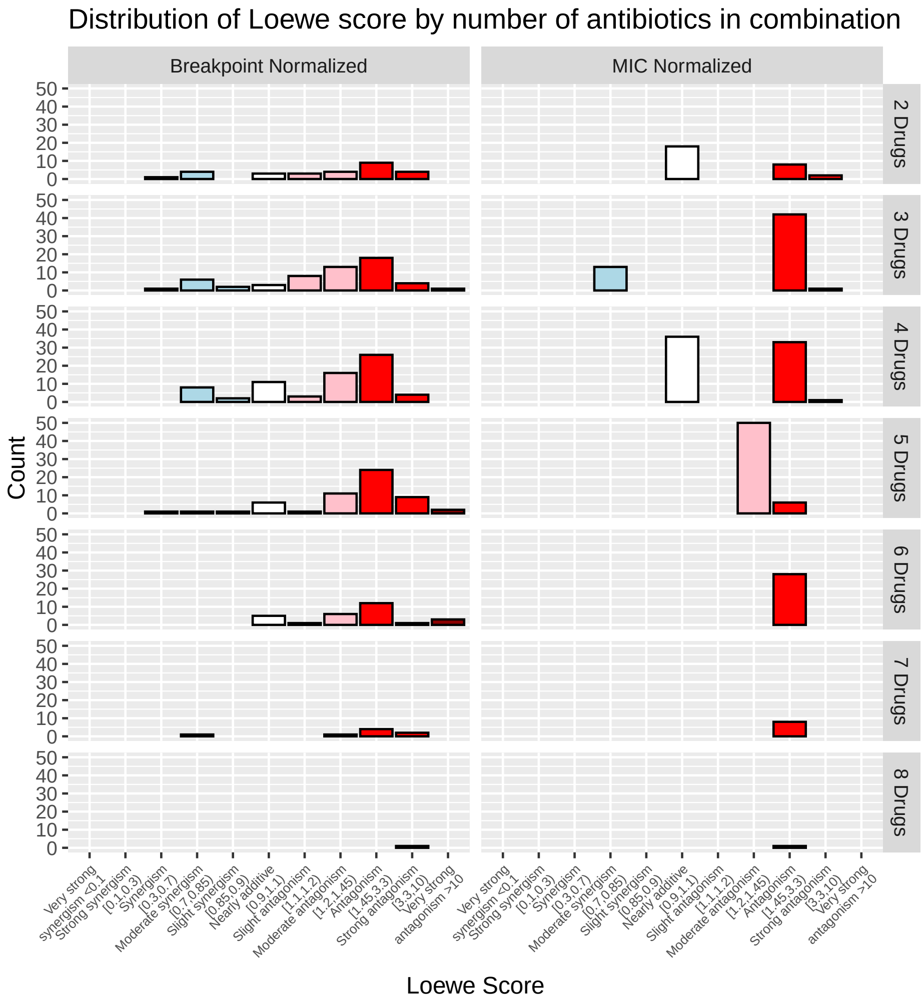

Figure A: Loewe interaction scores, calculated along the NDS diagonal, interpreted according to the criteria given in [1]. Observe that the two-fold discretization at which we sampled combinations leads to discretization in realizable Loewe scores, which is most apparent in the MIC-normalized experiment (right panel).

We emphasize that this only captures Loewe synergies for the combinations at the tested concentration ratios; it is possible that combining the antibiotics at different ratios would result in different Loewe scores. This concept is illustrated in Figure B, in which the choice of the diagonal on which to sample changes the determination of Loewe synergy.

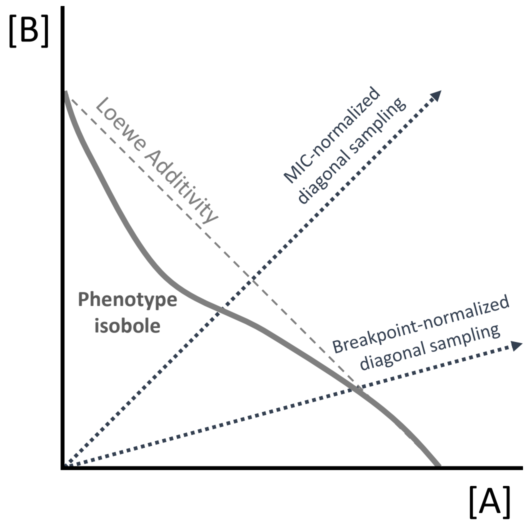

Figure B: Depending on the concentration ratio at which Loewe synergy is measured, different degrees of interaction may be reported. In this hypothetical example, sampling at the MIC normalized ratios results in a finding of stronger synergy than the breakpoint normalized ratio.

Figure C shows the Loewe interaction of drug combinations that exhibited "weak synergy" according to the Emergent Synergy Score, with the Loewe interaction determined along the concentration ratio sampled by the NDS design. Observe that the same combination may have a different Loewe synergy score across the two charts because it was measured at a different ratio in each experiment; this concept is illustrated in Figure B.

We see that most combinations that our method finds to be weakly synergistic are either synergistic or additive under the Loewe model, with only three combinations exhibiting Loewe antagonism. The Loewe perspective may also clarify why so many more combinations appeared to be synergistic under the MIC normalization: over 40% (18/44) of these weakly synergistic combinations were drug pairs that were effective when combined at half of their MIC. Such a combination is completely additive under Loewe, but is classified as weak synergy under our metric. The breakpoint experiments, by contrast, did not usually combine antibiotics at precisely half their MIC (since the breakpoint and MIC normalizations differ); as a result, pairs typically needed to exhibit Loewe synergy in order to exhibit synergy according to the ESS. We conclude by recalling that each diagonal was sampled on a twofold dilution gradient, which discretizes the value of Loewe synergies we might observe; if we were to repeat this experiment with a finer discretization, it would increase the precision of the Loewe synergy values.

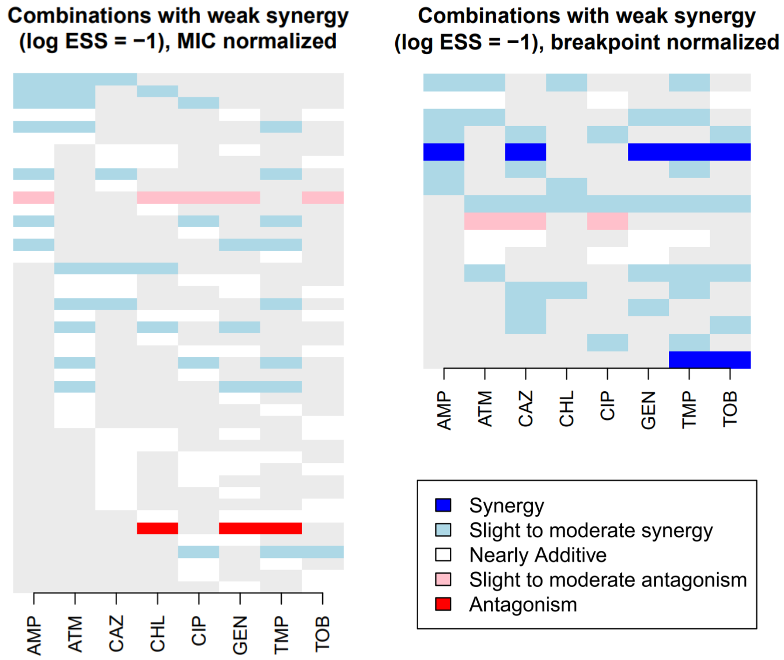

Figure C: Loewe synergy determination for the combinations that were determined to have weak synergy ($\log_2 ESS = -1$) according to our metric. Compare to Figures 2b and 3b; here the "present" antibiotics are colored according to the combination's Loewe interaction.

References

[1] Ting-Chao Chou. "Theoretical basis, experimental design, and computerized simulation of synergism and antagonism in drug combination studies". In: *Pharmacological reviews* 58.3 (2006), pp. 621–681.
