# Supplementary material for: Sample-efficient identification of high-dimensional antibiotic synergy with a normalized diagonal sampling design
Source: PLoS Comput Biol. 2022 Jul 18;18(7):e1010311. doi: 10.1371/journal.pcbi.1010311 (PMC9333450; doi:10.1371/journal.pcbi.1010311)
Supplement: S1 Data and Code — (ZIP) [file pcbi.1010311.s005.zip › Paper_supplement_data_code/MicNormalized/analysis/effectDist_withBackgroundColor.pdf]

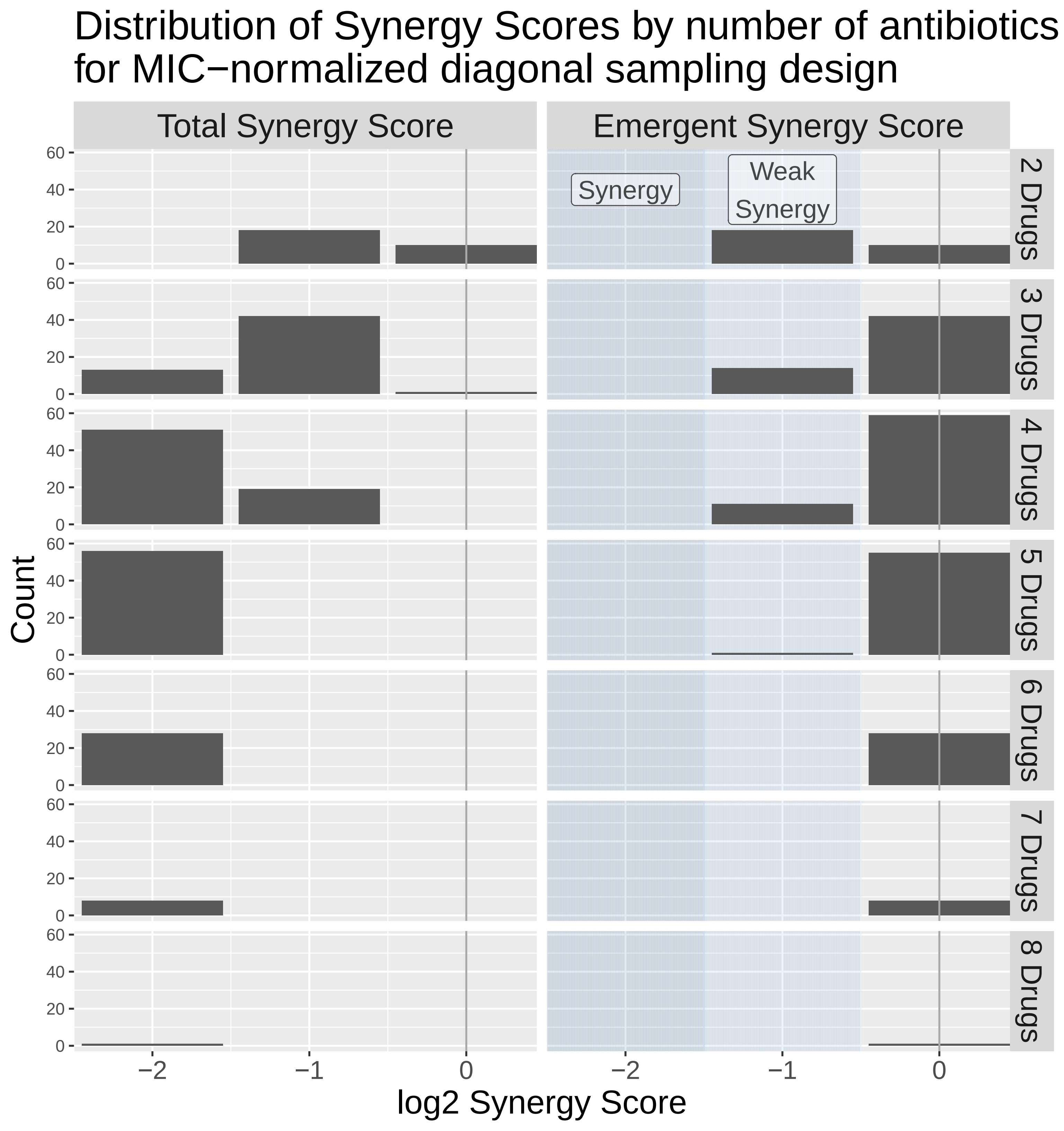

Distribution of Synergy Scores by number of antibiotics
for MIC−normalized diagonal sampling design
Total Synergy Score
Emergent Synergy Score
Synergy
Weak
Synergy
2 Drugs
3 Drugs
4 Drugs
5 Drugs
6 Drugs
7 Drugs
8 Drugs
Count
0
20
40
60
−2
−1
0
log2 Synergy Score
