# Supplementary figures and images for: Sample-efficient identification of high-dimensional antibiotic synergy with a normalized diagonal sampling design
Source: PLoS Comput Biol. 2022 Jul 18;18(7):e1010311. doi: 10.1371/journal.pcbi.1010311 (PMC9333450; doi:10.1371/journal.pcbi.1010311)

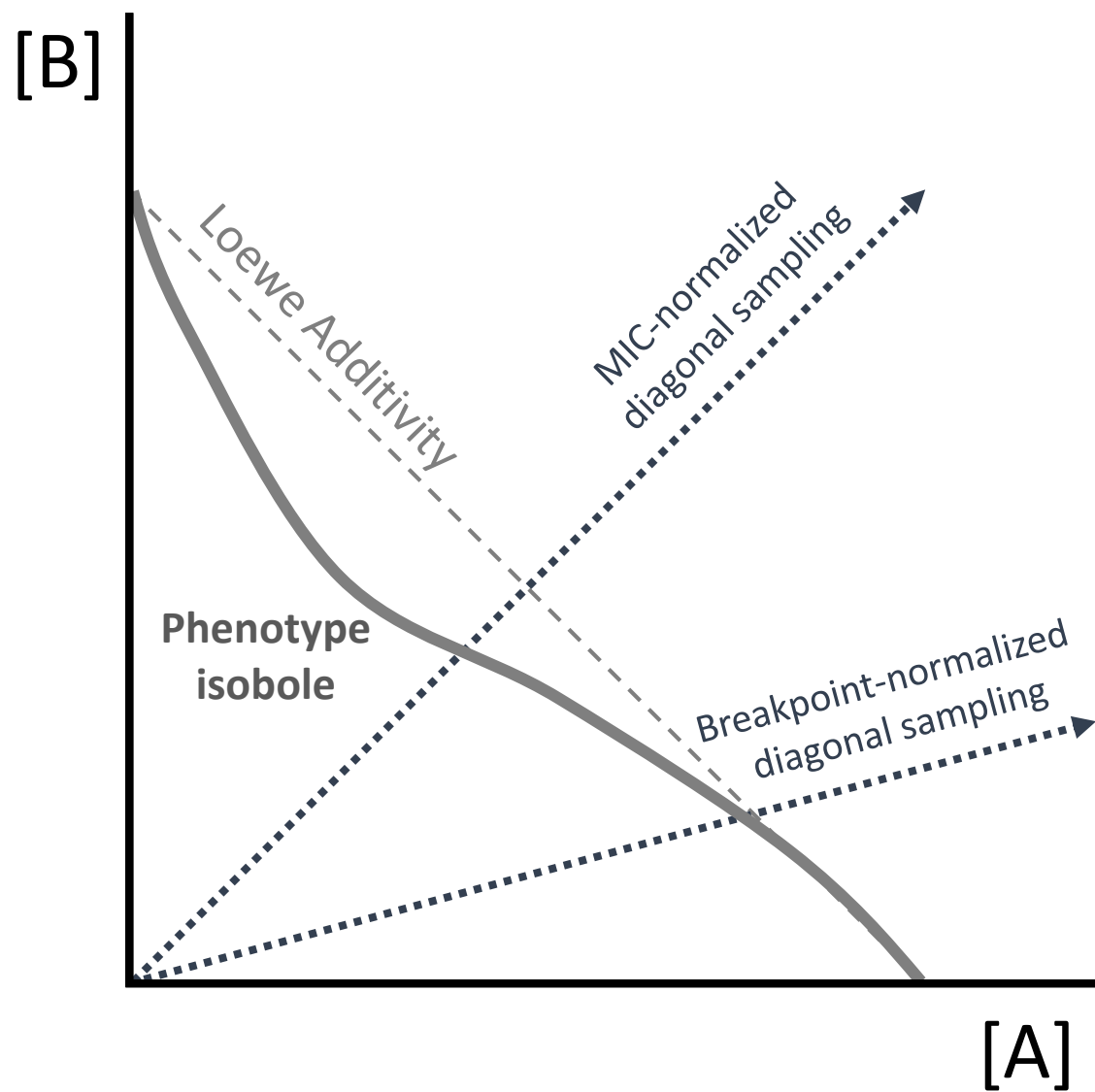

Supplement: S1 Data and Code — (ZIP) [file pcbi.1010311.s005.zip › Paper_supplement_data_code/LoeweAppendixFigures/LoeweIllustration.pdf]
