# Supplementary material for: Sample-efficient identification of high-dimensional antibiotic synergy with a normalized diagonal sampling design
Source: PLoS Comput Biol. 2022 Jul 18;18(7):e1010311. doi: 10.1371/journal.pcbi.1010311 (PMC9333450; doi:10.1371/journal.pcbi.1010311)
Supplement: S1 Text — (PDF) [file pcbi.1010311.s006.pdf]

Contents of Supplementary Code and Datasets Folder

This folder contains the (derandomized) raw data, AUGC, and rate measurements for each replicate of the 256 conditions tested in both the breakpoint-normalized and MIC-normalized experiments.

There is one subfolder for each experiment. They each contain the following:

- CSV files containing the (derandomized) raw OD readings, AUGC, and rate for each well.
- A CSV file identifying individual wells that should be removed from the analysis, due to observations or mistakes made during the experiment
- Commented R code to compute the MECI, TSS and ESS from the provided data and generate the plots in the paper
- An output CSV file ("`SynergyScores-*.csv`") identifying the MECI, TSS and ESS for all drug combinations. Also computes the Loewe synergy along the diagonal tested.
- A folder, "`analysis`", containing the plots generated by the R script

In addition, the file "`Comparisons.R`" generates plots that compare the two normalizations. This includes a comparative Loewe analysis (S4 Appendix) as well as a visualization of the combinations exhibiting weak synergy that includes information about the agreement between normalizations.

Finally, the folder "`NonparadoxicalGrowth`" contains the data from S3 Appendix, experimental spot-checking of the non-paradoxical growth assumption by testing 100 random dose-response curves in high-dimensional antibiotic space.
