# Supplementary material for: Sample-efficient identification of high-dimensional antibiotic synergy with a normalized diagonal sampling design
Source: PLoS Comput Biol. 2022 Jul 18;18(7):e1010311. doi: 10.1371/journal.pcbi.1010311 (PMC9333450; doi:10.1371/journal.pcbi.1010311)
Supplement: S1 Data and Code — (ZIP) [file lowestEmergence.pdf › Paper_supplement_data_code/BreakpointNormalized/analysis/lowestEmergence.pdf]

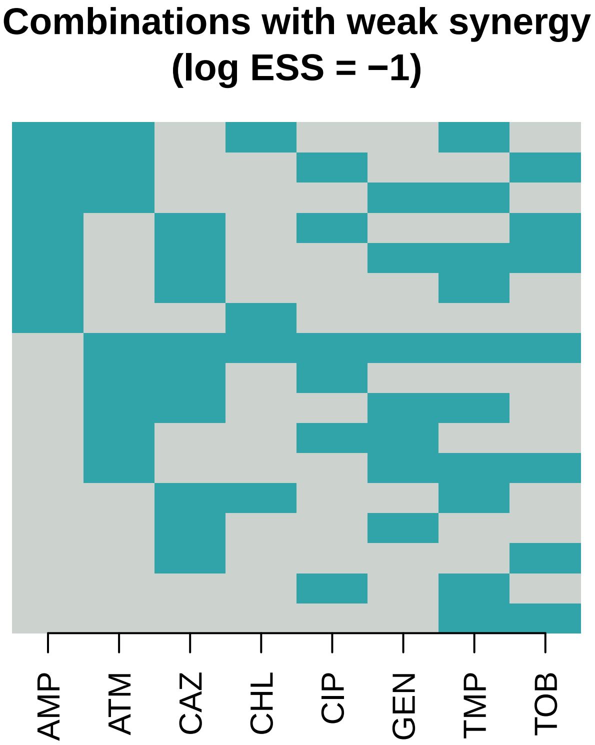

Combinations with weak synergy
(log ESS = −1)
AMP
ATM
CAZ
CHL
CIP
GEN
TMP
TOB
